# The Country Woman

**Published:** 1984-01

**Authors:** 


### THE COUNTRY WOMAN

There's nothing the matter with me
I'm as healthy as I can be.
I have arthritis in both my knees
And when I talk I speak with a wheeze.
My pulse is weak and my blood is thin,
I'm awfully well for the shape I'm in.
Arch supports I have for my feet
Or I wouldn't be able to be on the street.
Sleep is denied me night after night
But every morning I find I'm, all right.
My memory is failing, my head's in a swim
I'm awfully well for the shape I'm in.
For you and me who are growing old
The moral is this as my tale I unfold.
It's better to say 'I'm fine' with a grin
Then to let folks know the shape we're in.
How do I know that my youth is spent?
Well, my get up and go, has got up and went.
But I really don't mind when I think with a grin
Of all the places my 'get up' has 'bin.
Old age is gold, I've heard it said,
But sometimes I wonder as I get into bed
With my ears in a drawer, my teeth in a cup,
My eyes on the table until I wake up.
E'er sleep overtakes me I say to myself
Is there anything else I can lay on the shelf?
When I was young my slippers were red
I could kick my heels right over my head.
When I was older my slippers were blue
But still I could dance the whole night through.
Now I am old my slippers are black
I walk to the store and puff my way back.
I get up each morning and dust up my wits
Pick up my paper and read the 'Obits'.
If my name is still missing, I know I'm, not dead,
So I have a good breakfast and go back to bed.

Anon.
Submitted by D. W. W.

